# Hybrid VMAT-3DCRT as breast cancer treatment improvement tool

**DOI:** 10.1038/s41598-023-50538-x

**Published:** 2024-01-03

**Authors:** Cyril Voyant, Morgane Pinpin, Delphine Leschi, Séverine Prapant, Françoise Savigny, Marie-Aimée Acquaviva

**Affiliations:** 1 SPE Laboratory, University of Corsica, Corte, France; 2 Radiation Unit, Hospital of Castelluccio, Ajaccio, France

**Keywords:** Breast cancer, Biological physics

## Abstract

Radiation therapy is an important tool in the treatment of breast cancer and can play a crucial role in improving patient outcomes. For breast cancer, if the technique has been for a long time the use of 3DCRT, clinicians have seen the management evolve greatly in recent years. Field-in-field and IMRT approaches and more recently dynamic arctherapy are increasingly available. All of these approaches are constantly trying to improve tumour coverage and to preserve organs at risk by minimising the doses delivered to them. If arctherapy allows a considerable reduction of high doses received by healthy tissues, no one can deny that it also leads to an increase of low doses in tissues that would not have received any with other techniques. We propose a hybrid approach combining the robustness of the 3DCRT approach and the high technicality and efficiency of arctherapy. Statistical tests (ANOVA, Wilcoxon, determination coefficient, ROC, etc.) allow us to draw conclusions about the possibility of using the hybrid approach in certain cases (right breast, BMI $> 23$, age $> 48$, target volume $> 350$ cc, etc.). Depending on the breast laterality and patients morphological characteristics, hybridization may prove to be a therapeutic tool of choice in the management of breast cancer in radiotherapy.

## Introduction

Improving the quality of breast cancer treatment is essential to reduce mortality and radiation effects incidence in younger woman and to propose good outcomes after early tumour detection and an increasing of breast cancer Sequelae-free survival rates in general as breast cancer is one of the most common cancers in women of all ages^1^. The age of onset can vary, with a significant number of cases occurring in women over 50 but sometimes in younger women. Early detection and prompt treatment can lead to more positive outcomes, so continued efforts to improve breast cancer treatment and care are essential. Thanks to advances in technology, diagnosis and treatment methods, breast cancer survival rates have improved significantly. However, there is still much room for improvement, and ongoing research, clinical and dosimetric trials are essential to advancing the quality of care.

### Radiation therapy role in breast cancer

Radiotherapy is an important tool in the treatment of breast cancer and can play a crucial role in improving patient outcomes^2^. One of the main advantages of radiotherapy is its ability to target and destroy cancer cells while minimising damage to healthy tissue^3^. This makes it an effective option for treating breast cancer, particularly when used at an early stage^4^. Intensity modulated radiation therapy (IMRT), volumetric modulated arc therapy (VMAT) and three-dimensional conformal radiation therapy (3DCRT) are all forms of radiation therapy used in the treatment of breast cancer^5^. IMRT is known for its ability to deliver high doses of radiation to the tumour while minimising high dose exposure to surrounding healthy tissue^6^. This increases the accuracy and precision of cancer targeting and helps reduce side effects^7^. VMAT also delivers high doses of radiation to the tumour while minimising exposure of surrounding healthy tissue^8^ and has the advantage of a faster treatment time^9^. However, it is linked to a frequent increase in low doses in healthy tissues adjacent to the target volumes. 3DCRT is the reference and is a widely available and cost-effective form of radiotherapy, but it is less accurate and precise in targeting cancer than IMRT and VMAT, and results in greater exposure of surrounding healthy tissue^10^. It is important to note that exposure to high and low doses of radiation^11^, can increase the risk of damage to the heart^4^ and lungs^12^. Moreover the risk of second cancer induction on contralateral breast in particular for young woman must be considered like it is reported in literature^13^. Although less severe, the consequences and doses delivered to healthy mammary glands merit particular attention.

### VMAT and low doses (<10 Gy)

What is known since the introduction of VMAT in breast cancer, is that it is a fabulous technique but that it induces a considerable increase in the volume of healthy tissue receiving low doses. Many teams are wondering about the possible deleterious radiobiological effects of these doses. This exposure can have long-term effects on the lungs^14^, including a slight increase in the risk of lung cancer, fibrosis (scarring of the lung tissue) and impaired lung function. Studies have shown that exposure to low doses, can lead to changes in the DNA of lung cells that can result in mutations and an increased risk of lung cancer. It is important to note that the risk of lung damage from low-dose radiation exposure during radiotherapy depends on several factors, including the patient’s age, general health and the specifics of the radiotherapy such as dose, fractionation and irradiated volume. Several references support these conclusions, including^15^ and^16^. It should be noted that some papers suggest fatal lung disease as a function of low dose exposure after radiotherapy^17^, so it seems important to take these low doses into account and to propose new approaches accordingly. The same conclusions could be made by focusing on the effects on the heart as shown in Kang et al.^18^. Indeed, exposure of the heart to low-dose radiation can increase the risk of long-term cardiovascular effects, such as coronary heart disease and heart failure. The precise dose-response relationship for these effects is not well understood, but the risk is generally thought to increase with dose. It is likely that other organs are affected by low doses, but in the absence of literature it is best to apply the precautionary principle and try to minimise the use of techniques that induce low dose exposure to organs at risk. It is important to keep in mind that there is no direct evidence indicating that VMAT is not appropriate for breast cancer treatment, but it is important to use caution when using VMAT for large volumes of tissue that may receive low doses of radiation and to determine the best approach for each patient.

### The choice of hybrid approach (3DCRT and VMAT)

The advantage of models combination in engineering and physical sciences is that it allows the strengths of multiple models to be combined to overcome the limitations of a single model. Each model brings its own strengths and limitations, and their combination can provide a more complete view of the system and lead to more accurate and robust actions. It is in this perspective that it has been proposing for some years in radiotherapy, a hybridization of 3DCRT and VMAT models^19^. For breast cancer radiotherapy, a hybrid approach combining 3DCRT and VMAT may offer several advantages^20^. 3DCRT is a traditional and modulation-free technique inducing lower coverage of target nodes and may result in higher radiation doses to organs at risk with a volume of low doses in general, restricted. VMAT, on the other hand, uses intensity modulated arcs to deliver a radiation dose more consistent with target volumes (breast and nodes), reduces outside targets high doses but increases low doses. By combining these techniques, a hybrid approach can deliver a radiation dose more consistent with target volumes^21^, reduce doses to normal tissues^22^ and reduce the risk of long-term toxicity^23^. The decision to use a hybrid approach should be selective and reserved for certain patients^24^ and treatments^25^ with nodes irradiation for example^26^. An effort must be made on the simplicity, speed^27^ and quality of proposed treatments related to the best PTV coverage and OAR saving compromise^28^. The structure of this paper is classic with the next section relating to the presentation of data and planning methods (in “Material and methods” section), then a part which will deal with all the results (in “Results” section), before proposing a small paragraph dedicated to the technical feasibility of the hybrid method (in “Feasibility” section) and concluding (in “General conclusion” section).

The aim of the following demonstration is to quantify the contribution of the hybrid method, while remaining objective about its limitations. In order to don’t weigh down the paper, we have retained only VMAT With regard to reverse planning, which is currently the most widely used technique^29^. As we shall see, the 3DCRT approach studied is a form of intensity modulation (field-in-field) without inverse planning.

## Material and methods

The patient sample used and the methodology followed throughout the simulations will be detailed in the “Patient sample” and “Treatments planning” sections. Then we will expose the comparison metrics used to rank the three planning methods in “Comparison metrics” section.

**Table 1:** Description of patients enrolled in the study with Body Mass Index (BMI), age and volume of PTV target with 50 Gy dose prescription (Vol-PTV50 in cubic centimetre).

	Breast R & L (30 pts)			Breast R (15 pts)			Breast L (15 pts)		
	BMI	Age	Vol-PTV50	BMI	Age	Vol-PTV50	BMI	Age	Vol-PTV50
Mean	25.22	59.63	663.34	25.11	55.33	657.34	25.33	63.93	669.34
SD	4.82	17.24	426.49	5.20	18.33	470.72	4.58	15.48	393.86
Median	24.90	61.00	500.43	24.90	54.00	521.35	24.90	62.00	455.80
max	37.46	90.00	1910.00	37.46	90.00	1910.00	34.60	88.00	1541.00
min	16.30	25.00	151.90	16.30	25.00	151.90	19.00	42.00	257.60
Kurtosis	0.61	-0.71	1.25	1.85	-0.70	2.33	-0.49	-1.10	0.25
Skewness	0.60	0.03	1.26	0.85	0.14	1.44	0.33	0.25	1.14

### Patient sample

**Figure 1:**
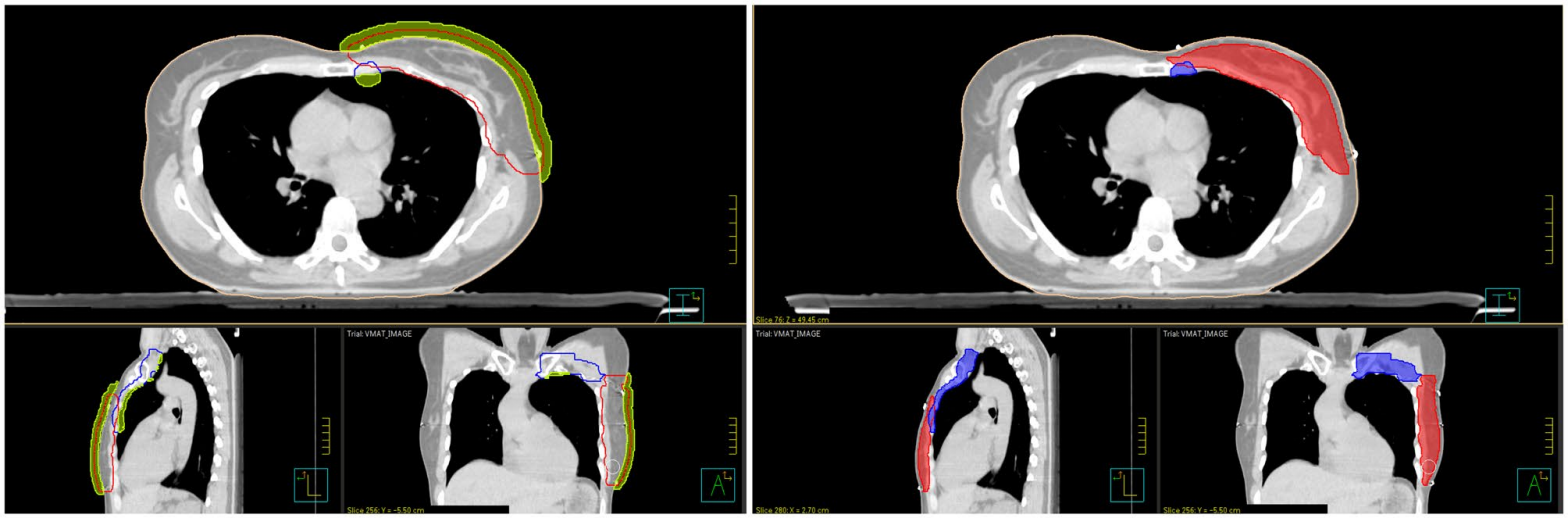
Target volumes illustrations on the left PTV50 (red) PTV47 (blue) and bolus (yellow), on the right PTV50-Eval (red) and PTV47-Eval (blue).

To address the hybridization contribution concerning free breathing breast cancer treatments with nodal prophylactic irradiation (internal mammary chain, intrerpectoral, level 1–4 axillary and medial supraclavicular region), a cohort treated between 2021 and 2022, was used (a random draw was carried out excluding total mastectomy). This retrospective study includes 30 patients (50% right breasts and 50% left one). The descriptive statistics are given in the Table  1. For each patient, objective validation criteria had to be defined, as well as volumes related to dosimetric optimization. The modalities for generating these volumes were respected for all patients included in the study as illustrated in Fig. 1. In Table  2 are indicated the setup margins used for the target volumes. The exhaustive list of all parameters used during this study for set-up, optimisation and validation are listed in Table  3. As an example, the clinical objectives are given, but it should be kept in mind that they do not serve as a reference since other criteria could be used. The clinical goals is 50 Gy (25 $\times$ 2 Gy) for the breast and 47 Gy (25 $\times$ 1.88 Gy) for nodes.

**Table 2:** Volumes definition ($X^C$ for the complement of the set *X*).

Volume	Definition	Remarks
PTV50	(CTV50 + 0.5 cm) $\cap$ (LungIL)$^C$	CBCT Reg/chest wall. Use for the optimization
PTV50-Eval	PTV50 $\cap$ (Ext-0.5 cm)	Use for the validation
PTV47	CTV47 + 0.5 cm	Use for the optimization
PTV47-Eval	PTV47 $\cap$ (Ext-0.5 cm)	Use for validation
Bolus breast^30^	(PTV50 + 0.5 cm) $\cap$ (Ext)$^C$	Density = 0.9 during optimization
Bolus nodes	PTV47	Density = 0.9 during optimization

**Table 3:** Dosimetric parameters definition. Note that PTV50 and PTV47 must imperatively be replaced by PTV50-Eval and PTV47-Eval if we had not simplified the labels for the sake of readability.

#	Structure	Parameter	Definition	Unit	Clinical goal
1	Target 50 Gy	PTV50-D98	Dose related to 98% of volume	Gy	45
2		PTV50-D2	Dose related to 2% of volume	Gy	53.5
3		PTV50-D50	Dose related to 50% of volume	Gy	50
4		PTV50-HI	Homogeneity Index^31^	unitless	0.14
5		Vol-PTV50	Volume of PTV50	cc	n/a
6		Vol-iso95	Volume of isodose 95%	cc	n/a
7		Vol-intersec	Vol-PTV50 $\cap$ Vol-iso95	cc	n/a
8		PTV50-CI	Conformity Index^31^	unitless	0.5
9		PTV50-V107	Volume of 107% of dose (53,5 Gy)	%	1
10		PTV50-V95	Volume of 95% of dose (47,5 Gy)	%	90
11		PTV50-V98	Volume of 98% of dose (49 Gy)	%	80
12	Tagets 47 Gy	PTV47-V95	Volume of 95% of dose (44,65 Gy)	%	95
13		PTV47-V98	Volume of 98% of dose (46,06 Gy)	%	80
14	Lung ipsolat	LungIL-Dmean	Mean Dose	Gy	15
15		LungIL-V20	Volume related to 20 Gy	%	30
16		LungIL-V30	Volume related to 30 Gy	%	20
17		LungIL-NTCP	NTCP related to Lyman model	%	5
18	Lung contralat	LungCL-Dmean	Mean Dose	Gy	5
19	Lungs (IL $\cup$ CL)	LungILCL-V5	Volume related to 5 Gy	%	50
20	Heart	Heart-Dmean	Mean Dose	Gy	5
21		Heart-V25	Volume related to 25 Gy	%	10
22		AIV-V30	Volume related to 30 Gy (AIV)	%	30
23	Liver	Liver-V5	Volume related to 5 Gy	cc	100
24	Breast contralat	BreastCL-Dmean	Mean dose	Gy	5
25	Humeral head	HH-Dmean	Mean dose	Gy	20
26	spinal cord (+3 mm)	PRVSP-Dmax	Max dose	Gy	20
27	Esophagus	Eso-V35	Volume related to 35 Gy	cc	5
28	Trachea	Trachea-V35	Volume related to 35 Gy	cc	5

AIV for Left Anterior Descending Artery or Anterior Interventricular Artery.

### Treatments planning

The plan followed in this study is relatively simple, we had, for each patient established three dosimetries (field-in-field based 3DCRT, VMAT and Hybrid) and several dosimetric parameters (listed in Table 3) were collected. IMRT was not included in this study since the results are close to those obtained with the field-in-field technique. The sample (30 patients) will allow to use different statistical metrics (defined in the following subsection) to conclude whether model combination is useful. Some rules were followed throughout the simulations. All contours (CTV & OAR) were equitably distributed between three physicians and plannings between two physicists. Note that thyroid is not included, even if it is associated to breast RT related toxicities and second cancer induction, because the statistical analysis revealed no significant difference between the 3 treatment methods studied. This organ is nevertheless integrated in volume PRVmed. The study guideline were:

- Isocentre positioned between supraclavicular nodes and the mammary gland in cranio-caudal direction and close to the center line in the internal-external one, so that realise CBCT is technically possible;
- Same isocentre for VMAT, 3DCRT and hybrid;
- Each trial (VMAT, Hybrid and 3DCRT) is done without comparison with the previous ones so as not to be tempted to enter into a logic of “fine-tuning” which would bias the study;
- Dynamic leaf gap of 1cm for all plans using arctherapy in order to improve plans quality control;
- VMAT plans made using the virtual bolus technique^32^;
- 3DCRT plans use different energies, wedge, fields-in-fields, etc. anything that allows to establish an acceptable output.

We insisted on proposing the most objective study possible by scrupulously respecting the rules set out above, as well as the definition of the beams that are identical for all patients and respects the characteristics set out in Table  4 (except for 3DCRT where considering patient’s anatomy, it may be useful to choose slightly different angle beam entrance, in order to better cover the target or spare OAR). The idea of reducing VMAT angles may seem attractive on regard the low doses contribution, but don’t forget that segment is updated every 2$^\circ$. This means that if we remove 20$^\circ$, 10 segments per beam are lost and therefore 30 segments for VMAT technique. Dosimetry becomes less efficient and ballistics more complicated (which reduces quality control validation). These angular limits are the consequence of 3 years’ experience with breast VMAT and are considered fixed. The characteristics of arcs used during VMAT and hybrid planning are detailed in Table  5. Simulations concern the Treatment Planning System Pinnacle (V16.4), two Elekta linacs (Synergy with MLC Agility) and free Breathing slow-CT acquisitions (BigBore Philips). Concerning the run$\#$1 of arcs optimization, the same process (Table  6 where PRVmed is defined by (larynx $\cup$ esophagus $\cup$ thyroid)+0.3cm) was applied for all patients. During next runs, planners modified PTV and OAR optimization criteria as desired^33^. To eliminate hot spots, most of the time, several passes (between 2 and 4) were necessary according to Algorithm 1 where No Man’s Land (NML) is defined from the isodose 107% ($\vee$ and $\wedge$ are respectively logical OR and AND).

**Algorithm 1:**
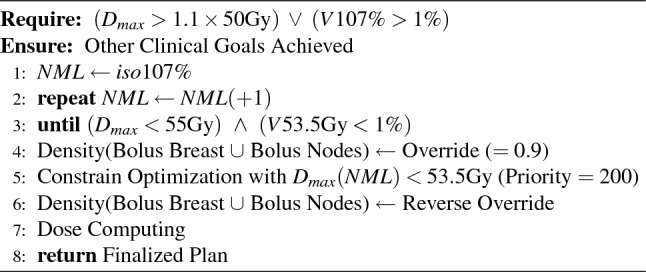
Hot Spots Removal

**Table 4:** Beams definition (IMN for internal mammary nodal region).

Method	Prescription	Beams	Weights (%)	Nature	Angle (R)	Rotation	Angle (L)	Coll
3DCRT	Breast+IMN	TGint	50	FiF (X6/18)	55	n/a	305	0
		TGext	50	Wedged (X6/18)	233	n/a	127	90/270
	Other	3 beams	Equal	{Wed.+FiF} (X6/18)	250/275/30	n/a	110/85/330	0/90/270
VMAT	Breast+Nodes	Arc1-1	25	VMAT (X6)	200/250	CC	290/350	0
		Arc1-2	25	VMAT (X6)	10/70	CC	110/160	0
		Arc1-3	50	VMAT (X6)	70/200	CCW	160/290	90
Hybrid	Breast+IMN	TGint	30	Open (X6)	55	n/a	305	0
		TGext	30	Wedged (X6)	233	n/a	127	90/270
	Breast+Others	Arc	40	VMAT (X6)	200/70	CCW	160/290	0

**Table 5:** Arcs definition (Pinnacle 16.4) for Elekta Synergy with Agility.

Method	Beam	Max. deliv. time (s)	Gant spac.	Seg. area	Leaf mot.	Conv. cycles	Cont. mod.	MU level	Dose fall off	Targ. falloff	TargEdge weight
VMAT	Arc1-1	50	2$^{\circ }$	15 cc	0,4 cm/$^{\circ }$	6/2	Med	Med	25	40	2
	Arc1-2	50									
	Arc1-3	100									
Hybrid	Arc	100									

The parameters are: maximum delivery time, gantry spacing, maximum segment area, the maximum leafs motion, the number of convolution cycles, the control modulation, the MU level, dose and traget falloffs ans the target edge weight.

**Table 6:** Optimization Initialyzing (run # 1).

Volume	Type	Target	Priority
PTV50	D95	47.5	Very high
	Uniforme dose	50.5	Defaut
PTV47	D95	44.65	Very high
	Uniforme dose	47.5	Defaut
PRVSP	Dmax	33	Very high
Lung ipso	Max EUD	12	Medium
Lung contra	Max EUD	5	Medium
Heart	Max EUD	5	Medium
AIV (anterior interventricular artery)	Max EUD	30	Medium
Breast contra	Max EUD	5	Medium
HH	Max Dose	43	Medium
PRVmed	Max EUD	30	Medium

Pinnacle’s Personnalized Planning doesn’t allow to quantify numerically the priority batches of the first optimization.

### Comparison metrics

Dosimetric comparison was carried out using conventional statistical tools. The more experienced reader will note that the normality of the distributions is not clearly established, necessitating non-parametric hypothesis testing. Wilcoxon rank sum and ANOVA test have been used to assess potential differences between data distributions related to the techniques used in this study. The use of coefficients of determination and correlation will complete the analysis, by making it possible to estimate the statistical link between dosimetric quantities. In the last part of the results, analyzes through receiver operating characteristic (ROC) curves will be proposed, testing if “a priori” factors (age, volume of 50 Gy target and BMI) could make it possible to highlight thresholds below which the clinical objectives (Table  3) are statistically achieved. The area of ROC curves (AROC) also referred to as area under curve (AUC) will be used to this task.

Results will be presented in order to compare the three planning methods presented above. We will start with probability distributions coupled with an ANOVA significance test^34^ (Part 3.1), then means comparison non-parametric test^35^ (Part 3.2), followed by coefficients of determination^36^ and a visual comparative approach (Part 3.3) preceding a comparison based on the rank correlation coefficient^37^ (Part 3.4). This is followed by a study of ROC curves^38^ (Part 3.5).

### Ethical approval and consent toparticipate

Clinical samples from patients were obtained after acquiring the informed consent of the patient in accordance with the protocol approved by Ethics Boards of Castelluccio Cancer Center and University of Corsica (rank 3 with IRB and RGPD). This study was performed in accordance with the Declaration of Helsinki.

## Results

Findings will be discussed in each part and then the main ones will be included in the conclusion. All the data and codes related to this study are available in https://github.com/cyrilvoyant/Hybrid.

### Probability density function

**Figure 2:**
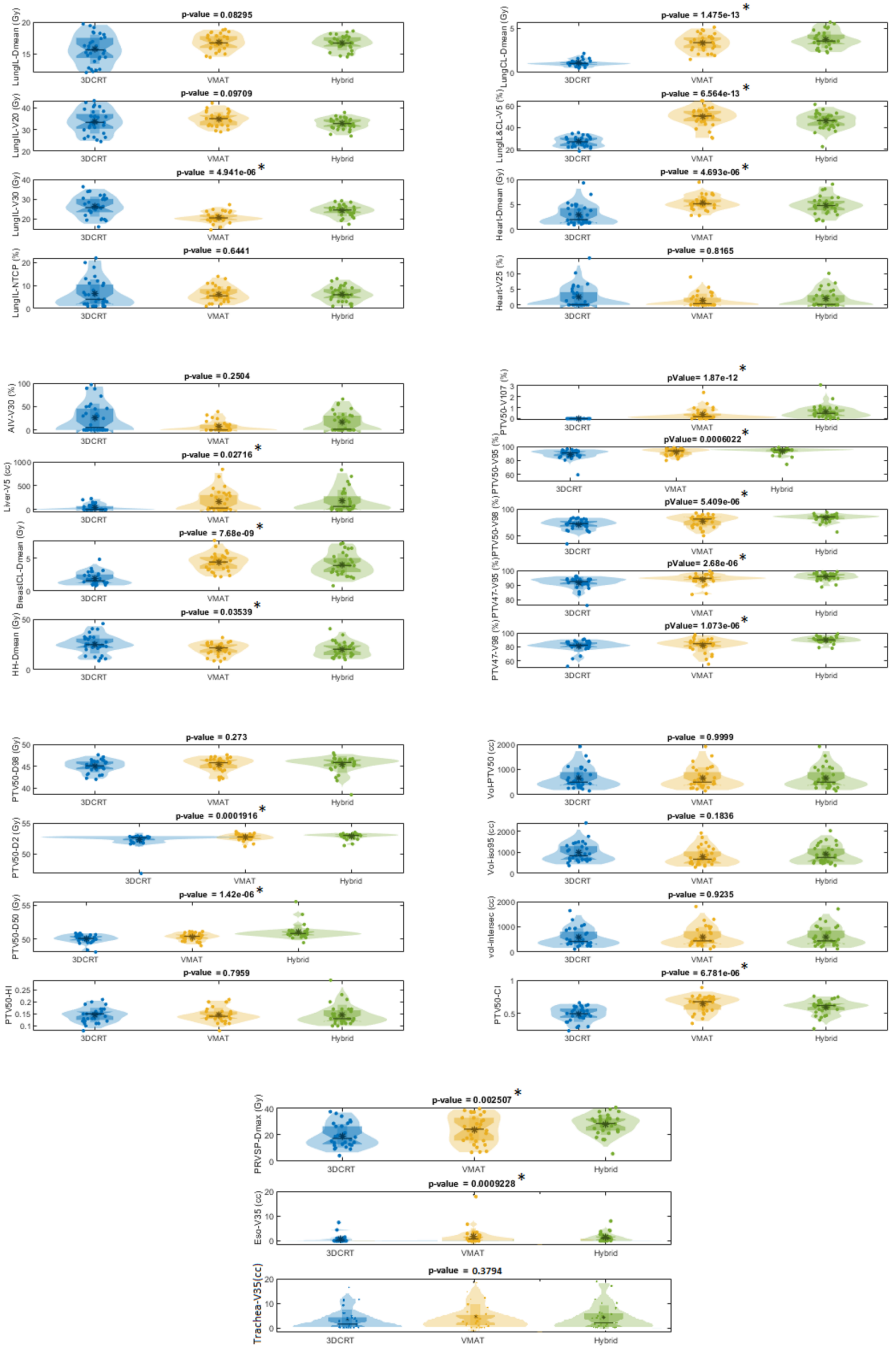
Violin plots of all parameters for the three planning methods and associated nonparametric one-way ANOVA on ranks test (an asterisk on side of plots with statistical significant i.e. *p*-value $<0.05$ induces that the 3 distributions are not related to the same population). Note that a Jarque-Bera test was operated and normality was rejected in over 70% of cases.

In Fig. 2 are represented the probability distributions (according to violin plots built with 30 patients) of parameters previously presented in Table 3. The three studied methods (3DCRT, VMAT and Hybrid) are considered. Results of non parametric one-way ANOVA (*p*-value) is shown in order to make easy the interpretation of the distributions comparison. The first important element that is visible is that the distributions are (mostly) not Gaussian, which leads to an inconsistent normal hypothesis. This is why we have favoured non-parametric statistical tools. Among the graphs that stand out, it is worth noting that for lungIL, only the V30 shows a significant difference between the three distribution types. The VMAT being the more suitable method showing good results. For LungCL and LungILCL, there is also an extremely significant difference highlighting the quality of the 3DCRT dosimetry. For the other parameters, there is nothing really conclusive and even if it is clear that the distributions are not from the same population (*p*-value$< 0.05$) the averages are relatively close and further studies are needed to conclude. In the following, we will separate three cases, all patients (n = 30), those who had treatment on the right breast (n = 15) and those on the left one (n = 15).

### Mean comparison and nonparametric test

**Figure 3:**
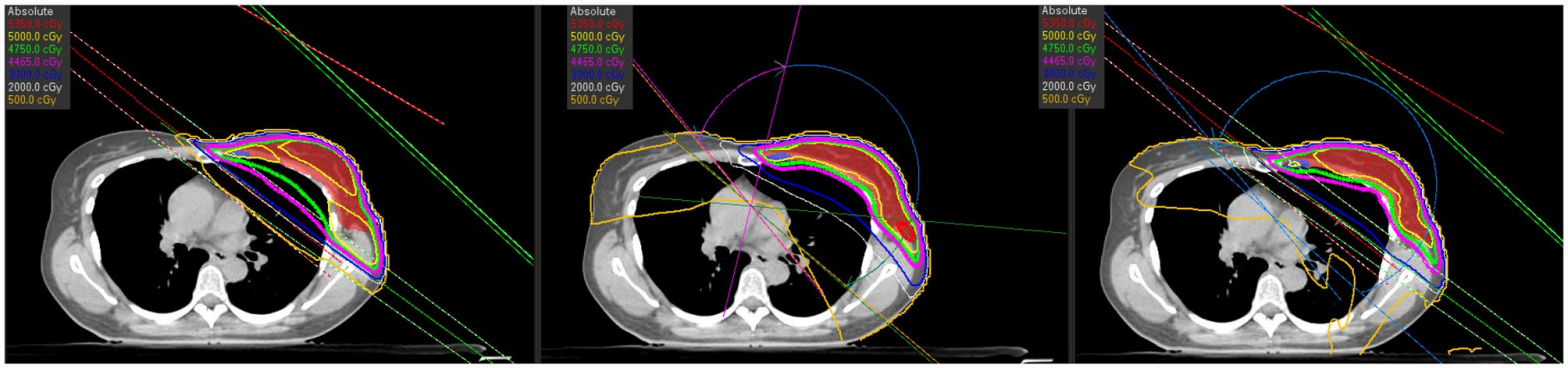
Isodoses and angle of beam related to the three treatments (from left to right : 3DCRT, VMAT and Hybrid).

The means of all parameters for all treatment techniques used are summarized in the Table  7. Both bold (best values) and italic (worst values) allow rank the three planning methods for each dosimetric variables (13 concerning the target volumes and 15 considering the organes at risk). At first sight, a trend seems to be emerging, if hybrid appears to be the method of choice for target volumes, 3DCRT is for organs at risk. On closer inspection, the differences are often not large, which leads us to use hypothesis testing to objectify the comparison. For ease but above all in order to make the comparison objective, we have decided to propose a non-parametric test on pairwise comparisons. This corresponds to three scenarios observed in Table  8. First, considering 3DCRT and VMAT, this last is the best way considering PTV50 and PTV47 for all the three samples. Mean dose related to LungIL and V5 of LungILCL are undeniably minimised by the 3DCRT method as shown in Fig. 3.

**Table 7:** Mean Values (bold for best results and italic for worst).

Parameters	Breast RL			Breast R			Breast L		
	3DCRT	VMAT	Hybrid	3DCRT	VMAT	Hybrid	3DCRT	VMAT	Hybrid
PTV50-D98	* 45.02*	45.38	** 45.4**	* 45.21*	45.93	** 46.03**	** 44.82**	** 44.82**	* 44.76*
PTV50-D2	* 52.3*	52.68	** 52.88**	* 52.48*	52.64	** 52.78**	* 52.11*	52.72	** 52.98**
PTV50-D50	* 50.01*	50.29	** 51.04**	* 49.95*	50.29	** 51.26**	* 50.07*	50.29	** 50.81**
PTV50-HI	** 0.145**	** 0.145**	* 0.146*	* 0.1447*	0.1327	** 0.13**	** 0.1453**	0.1573	* 0.1627*
Vol-PTV50	663.3	663.3	663.3	657.3	657.3	657.3	669.3	669.3	669.3
Vol-iso95	980.1	804.9	891.5	1004	812.1	910.8	956.6	797.7	* 872.2*
Vol-intersec	576.7	603	609.2	574	604.6	611.1	579.3	601.4	607.3
PTV50-CI	* 0.487*	** 0.645**	0.5917	* 0.471*	** 0.629**	0.578	* 0.504*	** 0.661**	0.6053
PTV50-V107	**0**	0.3923	* 0.612*	**0**	0.2967	* 0.6867*	**0**	0.488	* 0.5373*
PTV50-V95	* 88.94*	92.24	** 93.33**	* 89.4*	93.12	** 94.4**	* 88.48*	91.36	** 92.27**
PTV50-V98	* 70.91*	77.04	** 83.07**	* 70.28*	77.6	** 84.14**	* 71.53*	76.49	**82**
PTV47-V95	* 91.51*	94.03	** 95.73**	* 90.46*	94.64	** 95.39**	* 92.57*	93.41	** 96.06**
PTV47-V98	* 81.19*	82.49	** 90.06**	* 78.88*	82.91	** 89.22**	* 83.5*	82.06	** 90.91**
LungIL-Dmean	** 15.74**	* 16.86*	16.73	** 16.5**	* 16.93*	16.79	** 14.98**	* 16.79*	16.67
LungIL-V20	33.51	* 34.69*	** 32.63**	* 35.32*	34.15	** 33.36**	** 31.7**	* 35.23*	31.89
LungIL-V30	* 26.31*	** 20.85**	24.36	* 27.81*	** 20.58**	24.71	* 24.8*	** 21.11**	24.01
LungIL-NTCP	* 6.6*	6.3	** 6.267**	* 8.333*	** 6.667**	6.8	** 4.867**	* 5.933*	5.733
LungCL-Dmean	** 1.075**	3.357	* 3.736*	** 1.067**	3.274	* 3.981*	** 1.082**	3.44	* 3.49*
LungILCL-V5	** 26.94**	* 50.02*	45.99	** 30.67**	* 53.97*	50.51	** 23.21**	* 46.08*	41.47
Heart-Dmean	** 3.004**	* 5.378*	4.867	** 1.468**	* 4.501*	3.988	** 4.541**	* 6.255*	5.746
Heart-V25	* 2.502*	** 1.428**	2.049	**0.009**	* 0.052*	0.019	* 4.995*	** 2.803**	4.079
AIV-V30	* 25.25*	** 7.075**	16.88	**0**	**0**	**0**	* 50.49*	** 14.15**	33.75
Liver-V5	** 43.16**	172	* 178.8*	** 85.74**	310.9	* 317.5*	** 0.576**	33.14	* 40.15*
BreastCL-Dmean	** 1.918**	* 4.405*	4.047	** 2.482**	* 4.448*	4.378	** 1.355**	* 4.362*	3.716
HH-Dmean	* 25.16*	20.46	** 20.1**	* 24.99*	19.59	** 18.75**	* 25.34*	** 21.33**	21.46
PRVSP-Dmax	** 19.21**	24.01	* 27.62*	** 24.14**	28.62	* 30.45*	** 14.28**	19.41	* 24.79*
Eso-V35	** 0.583**	* 1.728*	1.471	** 0.206**	0.888	* 1.229*	** 0.959**	* 2.568*	1.713
Trachea-V35	** 2.468**	* 3.545*	3.32	** 3.056**	4.227	* 4.673*	** 1.88**	* 2.862*	1.967

**Table 8:** Significant parameters and associated *p*-value related to Wilcoxon rank-sum nonparametric test. In brackets, the method giving the best result for each dosimetric parameter.

	**BreastR &L**		**BreastR**		**BreastL**	
3D versus VMAT	PTV50-CI(VMAT)	*p* = 1.0e−05	PTV50-CI(VMAT)	*p* = 0.004	PTV50-CI(VMAT)	*p* = 0.001
	PTV50-V107(3D)	*p* = 1.9e−10	PTV50-V107(3D)	*p* = 2.7e−05	PTV50-V107(3D)	*p* = 2.5e−06
	PTV50-V95(VMAT)	*p* = 0.008	PTV50-V95(VMAT)	*p* = 0.0114	LungIL-Dmean(3D)	*p* = 0.018
	PTV50-V98(VMAT)	*p* = 0.014	PTV50-V98(VMAT)	*p* = 0.036	LungIL-V20(3D)	*p* = 0.040
	PTV47-V95(VMAT)	*p* = 0.001	PTV47-V95(VMAT)	*p* = 0.001	LungCL-Dmean(3D)	*p* = 4.5e−06
	LungIL-Dmean(3D)	*p* = 0.039	LungIL-V30(VMAT)	*p* = 0.001	LungILCL-V5(3D)	*p* = 3.3e−06
	LungIL-V30(VMAT)	*p* = 3.5e−05	LungCL-Dmean(3D)	*p* = 5.0e−06	Heart-Dmean(3D)	*p* = 0.003
	LungCL-Dmean(3D)	*p* = 7.0e−11	LungILCL-V5(3D)	*p* = 3.3e−06	AIV-V30(VMAT)	*p* = 9.6e−05
	LungILCL-V5(3D)	*p* = 1.4e−10	Heart-Dmean(3D)	*p* = 3.3e−06	BreastCL-Dmean(3D)	*p* = 4.1e−06
	Heart-Dmean(3D)	*p* = 5.2e−06	Liver-V5(3D)	*p* = 0.005		
	Liver-V5(3D)	*p* = 0.029	BreastCL-Dmean(3D)	*p* = 0.001		
	BreastCL-Dmean(3D)	*p* = 1.5e−08	Eso-V35(3D)	*p* = 0.013		
	HH-Dmean(VMAT)	*p* = 0.033				
	Eso-V35(3D)	*p* = 0.002				
3D versus Hyb	PTV50-D2(Hyb)	*p* = 1.2e−05	PTV50-D2(Hyb)	*p* = 0.011	PTV50-D2(Hyb)	*p* = 0.001
	PTV50-D50(Hyb)	*p* = 8.3e−07	PTV50-D50(Hyb)	*p* = 0.001	PTV50-D50(Hyb)	*p* = 0.001
	PTV50-CI(Hyb)	*p* = 0.001	PTV50-CI(Hyb)	*p* = 0.006	PTV50-CI(Hyb)	*p* = 0.043
	PTV50-V107(3D)	*p* = 1.6e−11	PTV50-V107(3D)	*p* = 8.6e−06	PTV50-V107(3D)	*p* = 6.8e−07
	PTV50-V95(Hyb)	*p* = 0.001	PTV50-V95(Hyb)	*p* = 0.001	PTV50-V95(Hyb)	*p* = 0.027
	PTV50-V98(Hyb)	*p* = 7.0e−07	PTV50-V98(Hyb)	*p* = 3.3e−05	PTV50-V98(Hyb)	*p* = 0.004
	PTV47-V95(Hyb)	*p* = 2.3e−06	PTV47-V95(Hyb)	*p* = 0.001	PTV47-V95(Hyb)	*p* = 0.001
	PTV47-V98(Hyb)	*p* = 4.6e−07	PTV47-V98(Hyb)	*p* = 0.001	PTV47-V98(Hyb)	*p* = 0.001
	LungCL-Dmean(3D)	*p* = 3.0e−11	LungIL-V30(Hyb)	*p* = 0.025	LungIL-Dmean(3D)	*p* = 0.016
	LungILCL-V5(3D)	*p* = 2.8e−10	LungCL-Dmean(3D)	*p* = 3.3e−06	LungCL-Dmean(3D)	*p* = 3.3e−06
	Heart-Dmean(3D)	*p* = 0.001	LungILCL-V5(3D)	*p* = 3.3e−06	LungILCL-V5(3D)	*p* = 1.6e−05
	Liver-V5(3D)	*p* = 0.015	Heart-Dmean(3D)	*p* = 7.4e−06	Heart-Dmean(3D)	*p* = 0.040
	BreastCL-Dmean3D)	*p* = 2.3e−06	Liver-V5(3D)	*p* = 0.008	Liver-V5(3D)	*p* = 0.048
	HH-Dmean(Hyb)	*p* = 0.023	BreastCL-Dmean(3D)	*p* = 0.001	BreastCL-Dmean(3D)	*p* = 8.8e−05
	PRVSP-Dmax(3D)	*p* = 0.000	PRVSP-Dmax(3D)	*p* = 0.038	PRVSP-Dmax(3D)	*p* = 0.001
	Eso-V35(3D)	*p* = 0.001	Eso-V35(3D)	*p* = 0.001		
VMAT versus Hyb	PTV50-D50(Hyb)	*p* = 0.001	PTV50-D50(Hyb)	*p* = 0.003	PTV50-D50(Hyb)	*p* = 0.037
	PTV50-CI(VMAT)	*p* = 0.040	PTV47-V98(Hyb)	*p* = 0.007	PTV47-V95(Hyb)	*p* = 0.021
	PTV50-V107(VMAT)	*p* = 0.033	LungIL-V30(VMAT)	*p* = 0.001	PTV47-V98(Hyb)	*p* = 0.005
	PTV50-V98(Hyb)	*p* = 0.019			LungIL-V20(Hyb)	*p* = 0.005
	PTV47-V95(Hyb)	*p* = 0.016			LungIL-V30(VMAT)	*p* = 0.011
	PTV47-V98(Hyb)	*p* = 0.001			AIV-V30(VMAT)	*p* = 0.001
	LungIL-V20(Hyb)	*p* = 0.014			PRVSP-Dmax(VMAT)	*p* = 0.038
	LungIL-V30(VMAT)	*p* = 2.9e−05				

### Determination coefficient study

**Figure 4:**
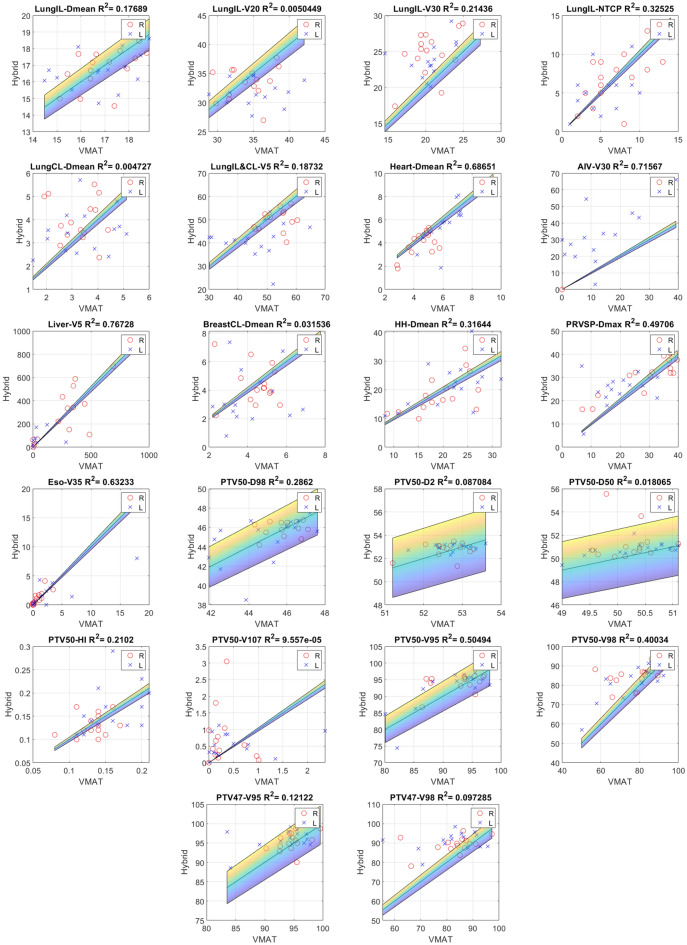
VMAT and Hybrid comparison for parameters where the determination coefficient ($R^2$) are lower than 0.8. Circles for right breasts and crosses for left one, the colored band corresponds to the non-significance area ($\alpha = 0.05$) so all points outside this band are significant. Points above the diagonal line signify a higher value fr Hybrid than for VMAT, and vice versa.

We have seen previously that VMAT and hybrid share common qualities and shortcomings regarding certain dosimetric parameters. This section will focus on separating these two types of approach proposing a graphical estimation using simple $y=x$ plots (Fig.  4). In addition, we suggest the use of coefficient of determination to refine the interpretation. Points (related to right or left breasts) above the diagonal line ($y=x$) signify a higher value for Hybrid than for VMAT, and vice versa. For points located in the colored band, no conclusion can be drawn as to its significance. For left breasts, V20 of LungIL are often in favour of hybrid, which is not necessarily true for right breasts. However, for V30 and AIV, the result is clear: VMAT puts everyone in agreement offering best results. For the mean dose of heart or dose related to PTV, Hybrid is preferable. It was chosen to show in this figure only results with an $R^2 < 0.8$ that is correspond to cases where a majority of points are outside the non-significance area. One parameter seems somewhat disturbing because its distribution of points seems random: LungCL. With an $R^2<0.01$ and a trend (especially for right breasts) in favour of VMAT (although below the clinical goal) few conclusions are possible.

### Spearman coefficient study

**Figure 5:**
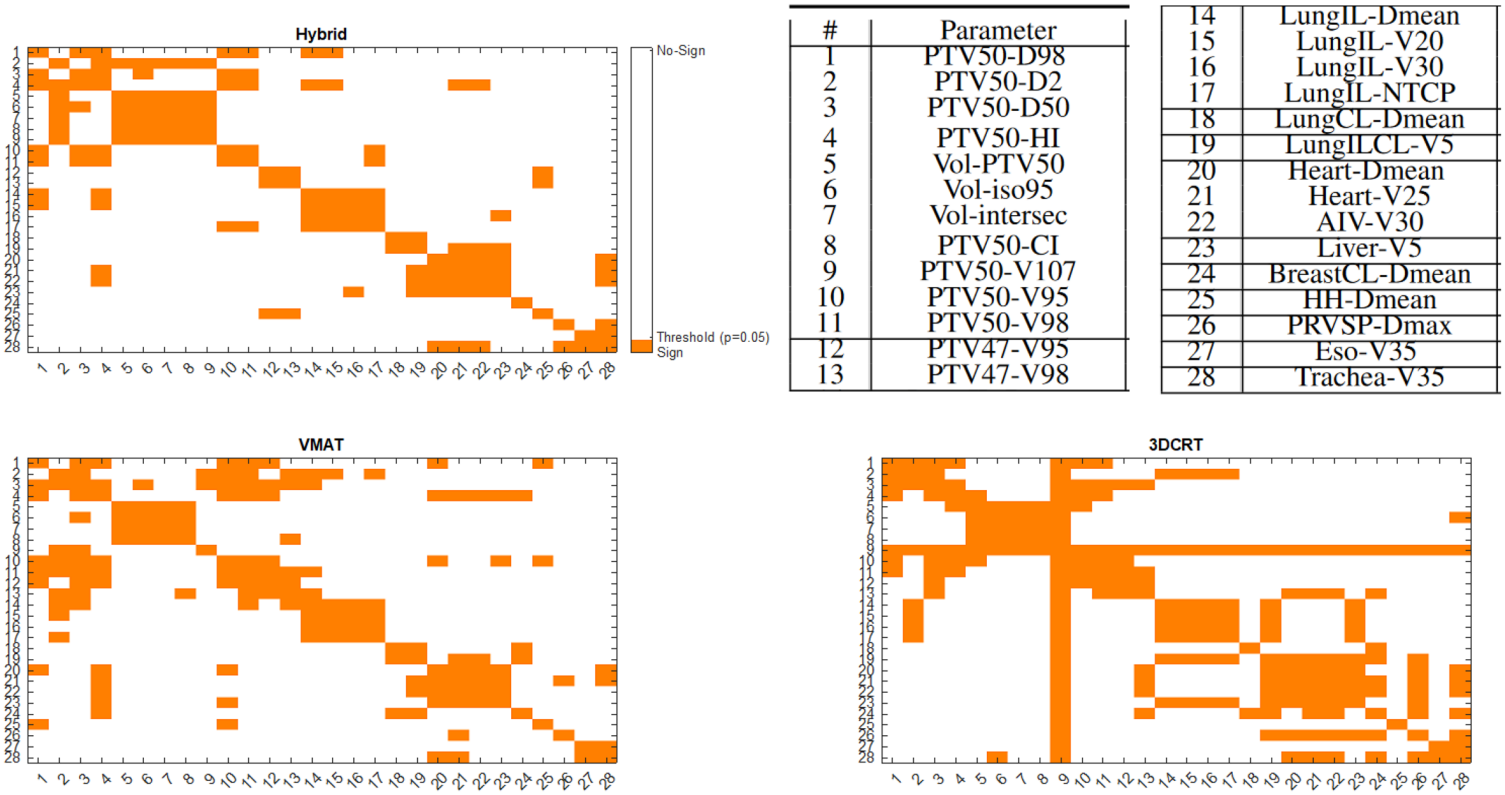
Spearman correlation analyze of all parameters (Table 3) and all treatment technics (Table 4). Colored boxes are related to a significant correlations ($\alpha =0.05$).

A cross correlation estimation is proposed concerning all available parameters. Figure  5 summarizes all the results related to the Hybrid, 3DCRT and VMAT plannings. The explanation related to the overall colored line observed with 3DCRT is because le PTV50-V107 is null for all patients. All significant correlations observed close to the diagonal are not surprising (no wonder the V95 and V98 of the target volumes are correlated), what will interest one, are the statistical dependencies outside the diagonals. Thus it is clearly seen that the AIV dose is strongly correlated with HI (for VMAT and Hybrid). This is important because it means to properly preserve the AIV, it is necessary to discover the target volumes. Even more true for the VMAT where HI low values are related to spared liver, heart, HH, etc. This phenomenon is also observed for the V95 of PTV50. For hybrid, it will mainly be the lungIL dose which will be correlated to the PTV50 coverage. There are fewer orange boxes for Hybrid than for VMAT outside the diagonal, suggesting that the Hybrid method is more robust and requires less compromise to use it.

### ROC study

**Figure 6:**
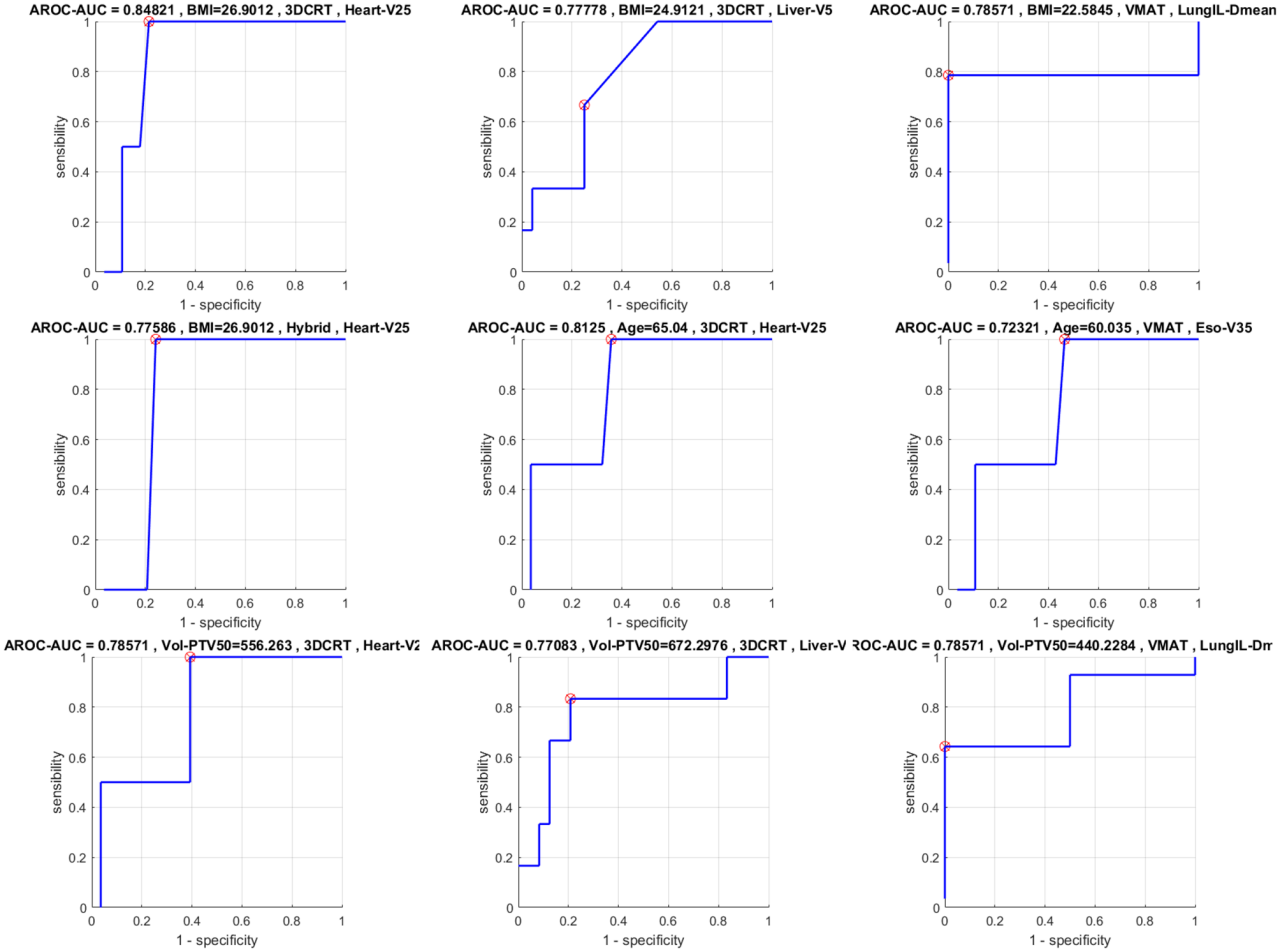
ROC Curves concerning $\text {AUC}>0.7$ for R &L Breasts. Red cross (and value in title of each plot) related to thresholds minimizing sensibility and specificity (=distance to point (0,1)) in order to pass the clinical goal test (Table 3).

**Figure 7:**
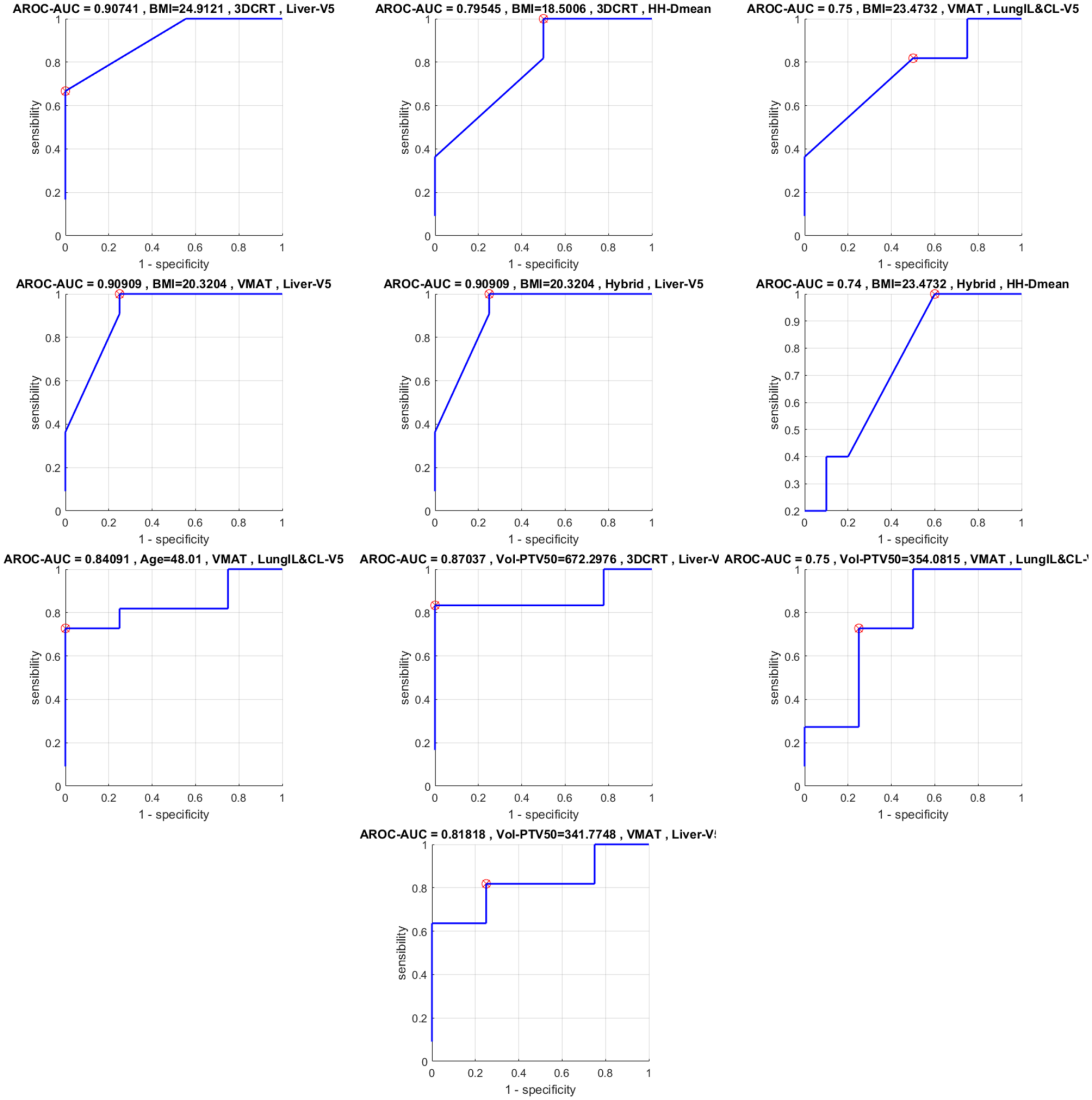
ROC Curves concerning $\text {AUC}>0.7$ for R Breast. Red cross (and value in title of each plot) related to the threshold minimizing sensibility and specificity (distance to point (0,1)) in order to pass the clinical goal test (Table 3).

As defined in the “Comparison metrics” section, this part is dedicated to the use of ROC curves. Only curves relating to AUC $>0.7$ will be presented so as not to make the reading of this paper cumbersome. This threshold, taken purely arbitrarily, corresponds in some way to a criterion of significance at 70%. In Fig. 6 are shown the results concerning both R &  L breasts while the Fig. 7 is dedicated to the R breasts. Note that there is not significant ROC curves concerning the L breast. In the first one, we see that age is very little represented. Let’s not forget that both sides are considered (right and left), so we will neglect Heart-V25 which is clinically irrelevant for right breasts. This leaves only an interaction between age and esophagus concerning VMAT which is surprising and probably not worth considering. The BMI and Vol-PTV50 are more relevant because for VMAT, BMI > 22.6 or Vol-PTV50 > 440cc induce a loss of chance to respect the LungIL-Dmean clinical goal (exposed in Table 3). No such result is obtained for hybrid but it is essential to compare this result with the average obtained in the Table 7 and which confirms this conclusion. If we focus on R breasts, the results are just as interesting. Indeed, VMAT induces a decrease in the success rate of clinical objectives concerning LungILCL-V5 when BMI > 23.4, age > 48 and Vol-PTV50 > 354. Again, 3DCRT and Hybrid outperform VMAT with a slight advantage for the latter, as it offers much better target volume coverage and therefore allegedly better tumour control. The thresholds established in this study should not be considered as absolute values. Any statistician will recognise statistical instability due to the small number of patients included in the study (15 R and 15 L). This is sufficient to draw conclusions on the trends observed but not to objectify thresholds or create dosimetric references. In the case of the present study, 50% left and 50% right would needs 384 data, considering a margin of error of 5% and an alpha level of 0.05.

### Feasibility

In this section, it is about how the treatments quality control (Delta4) make it possible to validate Hybrid approach generated during this study. It is important to propose a complete study which is not limited to a dosimetric one. The measurement aspect is important because the best theoretical technique could not be considered from a practical point of view, if it does not correspond to technical requirements. In our case, we use the local gamma passing rate criterion which must be $>95\%$ (concerning the 3%/3mm criterion and the threshold of 10%) as well as an average gamma lower than 0.4. In order to verify the dosimetric overlap between the two modes of dose delivery (static and arctherapy), we decided to test both arcs and tangentials (with a 9cm cranial offset to test as many points as possible). Results do not take into account the combined results (accumulation of several beams) but only results related to each beam in order to be objective and to limit the compensation effects. The gamma passing rate was equal for the first linac to 97.2% (std=1.23%) while for the second one it was 96.4% (std=1.67%). Note that the two linacs are identical (Synergy with Agility). The mean gamma was respectively 0.33 and 0.35 and the dose deviation 0.11 Gy and 0.16 Gy. This is in fact the same order of magnitude of what is observed during VMAT checks. Less than 5% of dosimetries fail to meet the previously mentioned technical goals and (sometimes) require to repeat dosimetric planning. It’s important to mention the impact of breathing on dose delivery. This can have a significant impact, particularly on different techniques with different delivery times (shorter or longer in relation to the respiratory cycle). In this sense, and in relation to Delta4 dosimetric assessments, one of the main limitations of this study is that we only measured the accuracy of plan deliverability, but not in vivo dosimetric verification.

### Brief summary of results

There are a number of results that can be highlighted from previous simulations. Below is a brief summary:

- For LungCL and LungILCL, there is an extremely significant difference highlighting the quality of the 3DCRT dosimetry;
- If hybrid appears to be the method of choice for target volumes, 3DCRT is for organs at risk;
- For V30 and AIV, the result is clear: VMAT puts everyone in agreement offering best results. For the mean dose of heart or dose related to PTV, Hybrid is preferable suggesting that it is more robust and requires less compromise to use it;
- 3DCRT and Hybrid outperform VMAT with a slight advantage for the latter, as it offers much better target volume coverage and therefore allegedly better tumour control;
- Note that pure multiple field IMRT technique with inverse planning is not considered as mentionned in the end of the introduction;
- In this study, we realize that the doses to the contralateral breast and lung are indeed low (Dmean < 10 Gy) but much higher than those observed with 3DCRT. The same is true for the average dose to the heart, which frequently exceeds 5 Gy. The strong points of VMAT are the reduction of high doses in organs at risk;
- Concerning D5 of the union of the two lungs or isolateral lung V20, everything suggests that VMAT is less efficient;
- For right breasts, it seems clear that Hybrid method is preferable, both in terms of lungs, contralateral breast and heart doses. This study reveals, however, that vigilance must be paid to spinal cord, trachea, esophagus and liver. Although the doses are low, it will be appropriate in the context of optimization to add a constraint concerning these organs (without necessarily a large weighting);
- In any case, we must not forget that these three methods are more complementary than rivals;
- It is important to mention that the increase of low dose bath (related to VMAT for example) will provide in principle an increase in second cancer induction, at different years post radiotherapy.

The final choice between the 3 methods (3D, VMAT and Hybrid) should be taken for each single scenario, and the conclusion of the presented study could only be taken as general indications and not rigid rules valid for each case.

## General conclusion

The methodologies and conclusions of this study support and improve those established in different studies dealing with hybridization in locoregional breast cancer treatment^39–41^. This study deals with the comparison between 3 types of ballistics used for the treatment of breast cancer. Note that pure multiple field IMRT technique with inverse planning is not considered as mentionned in the end of the introduction.. Among them, a hybrid approach mixing the robustness of 3DCRT and the high conformation of VMAT is examined. The idea that has been pointed out for some years concerning the use of arctherapy for such a pathology, lies in the fact of decreasing the low doses deposited in distant healthy tissues. Indeed, in this study, we realize that the doses to the contralateral breast and lung are indeed low (Dmean < 10 Gy) but much higher than those observed with 3DCRT. The same is true for the average dose to the heart, which frequently exceeds 5 Gy. The strong points of VMAT are the reduction of high doses in organs at risk, so that ipsolateral lung V30, IVA V30 and heart V25 are low with this type of treatment. Concerning D5 of the union of the two lungs or isolateral lung V20, everything suggests that VMAT is less efficient. It has been shown that patients classified as BMI > 23-23.5 or age > 48 or Vol-PTV50 > 350-450cc do not perceive a direct benefit from the use of VMAT due to increased lung doses and failure to achieve certain clinical goals (Tables 3 and 7). As a result, the hybrid method is positioned (and this was proven in this study) as a robust alternative to VMAT and 3DCRT. For right breasts, it seems clear that this method is preferable, both in terms of lungs, contralateral breast and heart doses. This study reveals, however, that vigilance must be paid to spinal cord, trachea, esophagus and liver. Although the doses are low, it will be appropriate in the context of optimization to add a constraint concerning these organs (without necessarily a large weighting). For the left breast, the question is more delicate. We would tend to favor the hybrid method, not least because of the PTV coverage. However, the VMAT and the resulting reduction in high doses to the heart do not allow us to make a clear decision (that’s no easy task with such a small sample). In any case, we must not forget that these three methods are more complementary than rivals. Therefore, if the material and human resources allow it, proposing three treatment plans to the physician seems the best solution so that he/she can decide according to what he/she prioritizes. The calculation tools allow to set up these ballistics and associated calculations quickly (< 2h), so it is not a real obstacle.

VMAT and hybrid QC show equivalent results in terms of gamma passing rate and mean gamma. If during this study, we have favored parsimony and proposed a hybrid approach with a single arc (in order to minimize the duration of the treatment), it is quite possible (and the dosimetric results are even better while the machine controls are equivalent) to propose a double arc approach (collimator angles set to 0$^{\circ }$ and 90$^{\circ }$) by keeping the limits of arm rotation identical to those presented here. It is likely that the hybrid approach will play an important role in the future of breast cancers treatment, even more so with deep inspiration breath hold technique that move the heart (for left-sided breasts cancer) away from the irradiated area. It is important to mention that the increase of low dose bath (related to VMAT for example) will provide in principle an increase in second cancer induction, at different years post radiotherapy^42^. The study of this risk is challenging, and maybe outside the intent of the present study. However, it is important to consider this important point^43^ for the future of breast cancer radiotherapy. Hybrid method could then be positioned as a highly attractive alternative. In this sense a second work will be initiated, where also the impact of imaging (e.g. daily or weekly, portal images or CBCT, partial or complete CBCT) may be included in the total dose estimation. This is crucial for young woman.

## Data Availability

The data that support the findings of this study are available from the corresponding author upon reasonable request.
